# Use of Virtual Reality in Psychiatric Diagnostic Assessments: A Systematic Review

**DOI:** 10.3389/fpsyt.2022.828410

**Published:** 2022-02-28

**Authors:** Chris N. W. Geraets, Märta Wallinius, Kristina Sygel

**Affiliations:** ^1^Lund Clinical Research on Externalizing and Developmental Psychopathology, Child and Adolescent Psychiatry, Department of Clinical Sciences Lund, Lund University, Lund, Sweden; ^2^Research and Development Unit, Regional Forensic Psychiatric Clinic, Växjö, Sweden; ^3^Department of Psychiatry, University Medical Center Groningen, Groningen, Netherlands; ^4^Department of Psychiatry and Neurochemistry, Centre for Ethics, Law and Mental Health, Institute of Neuroscience and Physiology, The Sahlgrenska Academy at University of Gothenburg, Gothenburg, Sweden; ^5^Department of Forensic Psychiatry, National Board of Forensic Medicine, Stockholm, Sweden

**Keywords:** virtual reality, assessment, diagnostic, psychiatry, mental disorder

## Abstract

**Background:**

Technological developments such as Virtual reality (VR) provide new opportunities to extend and innovate mental healthcare. VR as a tool for clinical assessment has been described as promising, as it can enable real-time assessment within real-like environments or contexts as opposed to self-report and behavioral tasks in laboratory settings.

**Objective:**

With this systematic review we aimed to provide an overview of recent studies using VR in the assessment of psychiatric disorders.

**Methods:**

A systematic search was performed using Pubmed, Embase, PsycInfo, and Web of Science between 2016 and 2020. Studies were included if they used immersive VR, concerned assessment of psychiatric symptoms/disorders, and included adult patients with psychiatric disorders.

**Results:**

The search resulted in 3,163 potentially eligible articles, from which a total of 27 studies fulfilled inclusion criteria. Most studies considered anxiety (*n* = 7), addictive, (*n* = 7), or psychotic disorders (*n* = 5). Regarding ADHD (*n* = 3), PTSD (*n* = 3), and pedophilic disorder (*n* = 1), a few studies had been performed since 2016. The majority of the included studies compared patient groups to healthy control groups.

**Discussion:**

Recent studies on VR-assisted psychiatric assessments have been conducted to validate VR environments, to assess symptoms for diagnostics or therapy goals, search for biomarkers, and to gain knowledge on psychiatric disorders. VR tasks were able to detect some difference between patient and healthy control groups, mainly with regard to self-report measures. Despite previous, promising prospects, the use of VR as a tool in clinical assessments must still be considered as a field in need of continued developments and evaluations.

**Systematic Review Registration:**

www.crd.york.ac.uk/prospero, identifier: CRD42021233772.

## Introduction

Assessments and the diagnostic process in psychiatry mainly consist of clinical interviews, self-report questionnaires, neuropsychological tests, and, in some cases, behavioral observations (1). Such assessments constitute a basis for the individual tailoring of psychiatric treatment for patients. However, interviews and self-report questionnaires strongly rely on patients' memory, current mental state, self-understanding, and insight. Also, the context within which psychiatric assessments are performed may be criticized for lack of ecological validity. Therefore, a need for ecologically valid psychiatric assessments with increasingly objective measures has been expressed, and method developments thereto related are needed (2).

Novel technologies, such as immersive virtual reality (VR), may provide new possibilities in the quest to fill this gap (3, 4). With immersive VR, patients enter computer-generated simulations, e.g., going to a grocery store, by using a head-mounted display or CAVE environment. Such immersive systems can deliver a fully surrounding, extensive and vivid illusion of reality to people's senses (5). VR simulations have been shown to trigger psychological and physical reactions similar to the reactions in real life (6). This feature—feeling real—makes VR a promising tool for psychiatric assessments (7). This perceived realness of a VR experience or the feeling of being “there” is called presence (8). Presence is the subjective illusion of being in a real place despite being physically located in a different place, causing people to react realistically to the VR (9). Furthermore, VR has the advantage that you can (repeatedly) expose patients to (social) situations that are completely controlled and can be accessed within a safe, clinical environment. Thus, the same VR social environment can be presented to multiple patients to assess, cognitions, behaviors, emotions, and physiological responses in real-time. Given this, VR-assisted psychiatric assessment has been described as promising as it may enable real-time assessments within (individually tailored) virtual environments or contexts that are perceived and experienced by the patient as “real.” This can create greater possibilities for increased ecological validity of psychiatric assessments as opposed to assessments relying on self-reports, interviews, and behavioral tasks conducted in laboratory settings (10, 11).

To date, two systematic reviews have investigated the existing evidence on VR-assisted psychiatric assessments up to 2016 (10, 12). van Bennekom et al. (12) identified 39 studies, mostly focusing on patients with a psychotic (*n* = 15) or developmental disorder (*n* = 12). The most common topics were paranoia and social behaviors. The authors concluded that almost all reviewed VR situations/scenarios enabled eliciting and measuring psychiatric symptoms to some extent. E.g., paranoid ideation has been measured in patients with psychotic disorders by exposing them to neutral VR environments (13, 14). The benefit of applying VR in such assessments lies in the fact that with self-report it is unknown whether reported hostility as experienced by the patient is real or not (15). In a controlled, VR environment with only neutral cues, a patient's self-reported level of perceived hostility in the VR environment then becomes easier to evaluate objectively. Supporting the validity of VR-assisted psychiatric assessments, van Bennekom et al. (12) found significant relations between VR measures and traditional diagnostic measures in 14 studies. However, relatively small groups were tested, limiting definite conclusions.

Similarly, Freeman et al. (10) concluded, in a broader systematic review on 285 studies (of which 86 considered assessments), that psychiatric symptoms can be assessed with VR, but that this at the time of the systematic review predominantly had been done to validate VR environments or gain an increased understanding of symptoms and mechanisms of psychiatric disorders, rather than for clinical assessment purposes. Also, the evidence for reliability and validity (e.g., convergent validity in relation to common self-report measures) for VR-assisted psychiatric assessments was demonstrated as very limited.

Over the past years, research on VR applications for mental healthcare has been rapidly expanding due to decreased costs and improved quality of software and hardware, and an update of the evidence-base for VR-assisted psychiatric assessments is needed. Therefore, this study aimed to provide a systematic review of recent studies (from 2016 onwards) applying immersive VR in psychiatric assessments. To this end, we reviewed VR assessment studies on differences between patients with a psychiatric disorder and healthy controls, and studies investigating the relation between VR measures and established diagnostic measures e.g., clinical assessments or patient-rated questionnaires of psychiatric symptoms and disorders.

## Methods

### Design

We conducted a systematic review on trials applying immersive VR in the assessment of psychiatric symptoms and disorders. This review was registered on PROSPERO (CRD42021233772). Two previous systematic reviews have described, in detail, the studies conducted up to 2016 (10, 12). This systematic review focuses on more recent findings reported between 01-01-2016 and 31-12-2020.

### Inclusion and Exclusion Criteria

Inclusion criteria were: (1) use of immersive VR (utilizing a head-mounted-display (HMD) or CAVE), (2) concerning assessment of psychiatric symptoms or disorders, (3) either comparing a patient group with a healthy control group or investigating the relation between a VR and an established measure, (4) concerning adult patients with a psychiatric disorder, (5) original empirical research findings (e.g., pilot, RCT, or case-series), (6) published in an English peer-reviewed journal, and (7) published between 01-01-2016 and 31-12-2020.

Exclusion criteria were: (1) letters to editors, theses, conference papers, or book chapters, (2) participants with primarily learning and intellectual disabilities, (3) full text unavailable, (4) unclarities concerning which VR technology was applied, and (5) one-to-one VR computerized version of a neuropsychological test.

### Literature Search and Study Selection

Four databases were searched: PubMed, PsychInfo, Web of Science, and Embase. See Supplementary Material 1 for the complete search strategies. For each of the following diagnostic/patient groups, specific search terms were created and added to the search: anxiety disorders, mood disorders, psychotic disorders, eating disorders, autism, ADHD, substance use disorders, impulse control disorders, sexual disorders, personality disorders, forensic psychiatry, and mental health generally. Databases were searched from 01-01-2016 up until 31-12-2020. The general keyword search terms were: Virtual reality OR “VR technology” AND assess^*^ OR diagnos^*^ OR test OR experiment OR evaluation AND—specific terms for each patient group.

First, duplicate references were removed and titles and abstracts were read to screen for relevant studies. Then, two researchers independently reviewed the full texts to determine whether the inclusion and exclusion criteria were met. Consensus between the researchers (CG and KS) was a prerequisite for inclusion, differences in judgment were solved by discussion between the two researchers.

### Quality Assessment

The Quality Assessment Tool for Quantitative Studies (QATQ) of the Evaluation of Public Health Practise Project was used for quality assessments of the included studies (16). Studies were rated on six domains: selection bias, study design, confounders, blinding, data collection methods, withdrawals and drop-outs. The QATQ provided a global rating based on the subscales: strong = no weak ratings, moderate = one weak rating, or weak = two or more weak ratings on the subscales.

## Results

**Figure 1:**
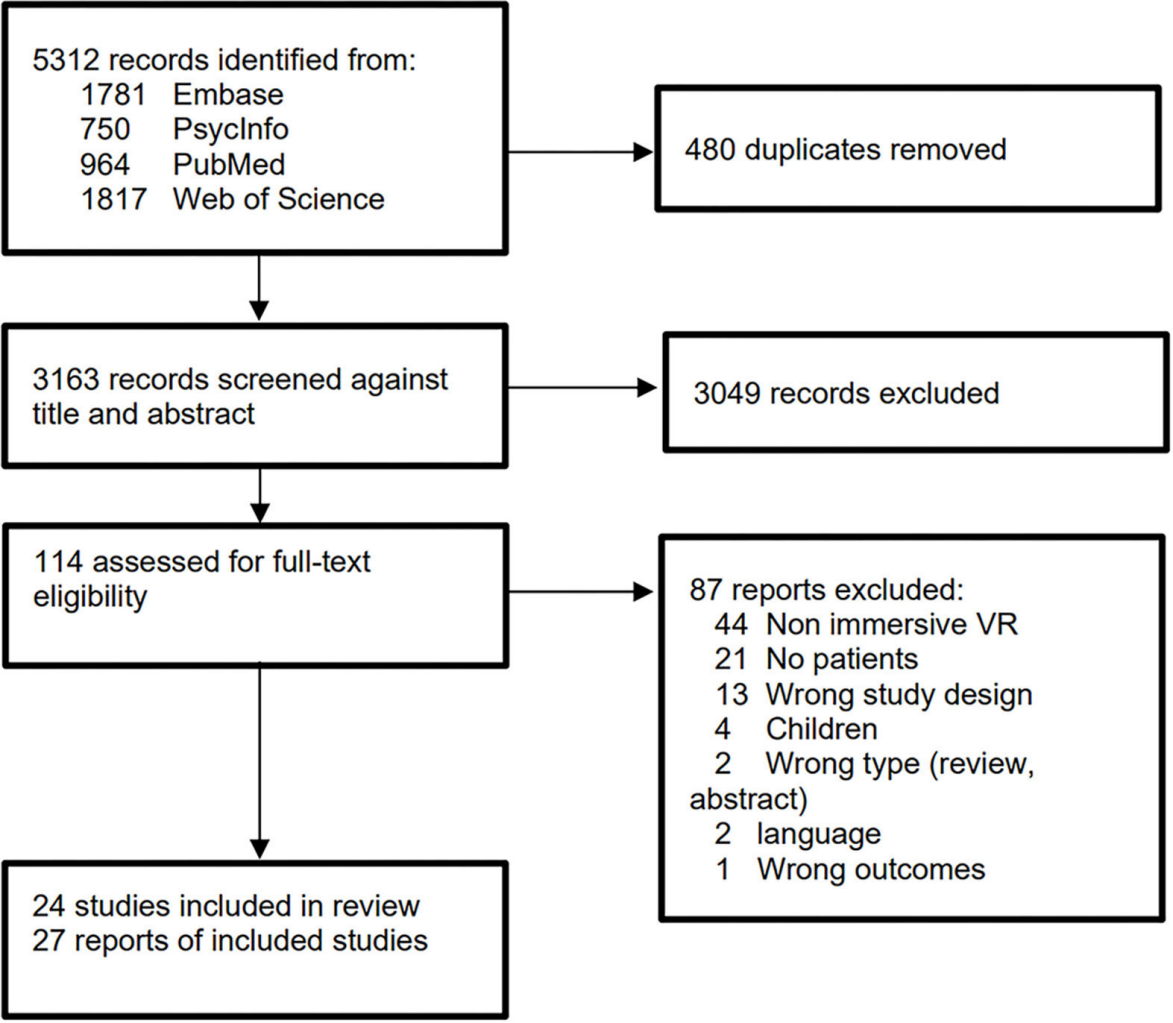
Flow diagram of the study inclusion process.

The final search was conducted on 23-02-2021 and, after duplicate removal, this resulted in 3,163 identified articles. In total, 27 articles met the inclusion criteria, see Figure 1 for a flowchart of the search. Two of the 27 studies concerned a follow-up publication to an original study (17, 18) that was also included. In total, these studies included 2,121 participants, of whom 1,123 were patients with psychiatric disorders and 998 were healthy controls. The most common reasons for studies not to be included during the full-text screening procedure were the use of non-immersive VR (e.g., use of 2d computer screens/desktops) and lack of a psychiatric patient group in the study.

Table 1 provides a summary of the included studies, presented per diagnostic subcategory. Most studies were performed on assessment of anxiety (*n* = 7), addictive (*n* = 7), or psychotic disorders (*n* = 5). Concerning, ADHD (*n* = 3), PTSD (*n* = 3), and pedophilic disorder (*n* = 1), only a few studies have been performed since 2016. Table 2 presents an overview of the results of the quality assessment for each study. Ten studies were of moderate quality, and 17 demonstrated a strong quality rating.

**Table 1:** VR-assisted psychiatric assessments.

**Study**	**Participants**	**Duration (min)**	**Topic and VR task**	**Main measures during and about VR**	**Main findings**
**ADHD**					
Camacho-Conde et al. (19), Spain	60 ADHD (0%♀) 60 HC (0%♀)	18	***Attention:*** Perform 1 learning and 2 dual execution tasks in the Nesplora Aquarium, subjects need to press/inhibit to press a button when they see specific fish or hear certain words.	*During VR:* correct answers, omission errors, commission errors, reaction time	ADHD-participants showed less correct answers, more omission errors, and slower reactions times, but not more commission errors, than HC.
**Pedophilic disorder**					
Fromberger et al. (20), Germany	6 pedophilic sexual offenders (0%♀) 7 healthy controls (0%♀)	20	***Risk situation coping:*** During 4 tasks, navigate a supermarket and buy products. When encountering an avatar asking for help, choose between multiple-choice options representing approach or avoidance behaviors. In task 1, subjects encounter an unattractive virtual person (control) and in task 2–4 an attractive virtual child (risk scenario).	*During VR:* coping in terms of approach/ avoidance behavior *About VR:* therapist and offenders predicted coping and behavior	Approach/avoidance responses differed in one risk scenario between HC and offenders. Offenders behaved incongruent to therapy-learned coping strategies in 50% (therapist-rated), and 62% (offender-rated) of the cases. Therapists predicted offenders' responses correctly in 75%.
**PTSD**					
van't Wout et al. (21), USA	19 PTSD veterans (0%♀) 24 non-PTSD veterans (4%♀)	12	***Biomarker PTSD:*** Watch 6 combat-related scenarios with increased intensity (e.g., encountering enemy fire) including olfactory and haptic stimulation, and watch 6 classroom scenarios with increased complexity.	*During VR:* skin conductance	PTSD veterans had higher skin conductance than non-PTSD veterans during VR combat events, but not in non-combat classroom scenarios. No relation between skin conductance and PTSD symptoms was found.
Ridout et al. (17), USA	19 PTSD veterans (0%♀) 19 non-PTSD veterans (4%♀) *Subsample van't Wout et al. (21)*	12	***Biomarker PTSD:** Same as van't Wout et al. (21) above*.	*During VR:* HRV	No difference in HRV was found between PTSD and non-PTSD veterans. No difference was found in HRV between the combat and classroom scenarios.
Malta et al. (22), USA	11 PTSD veterans 21 non-PTSD veterans Total (16%♀)	8	***Biomarker PTSD**:* Navigate and perform tasks in an Iraq city: search for insurgents, take cover in a marketplace during an attack, remain the search for insurgents after the attack ends. Stressors included gunfire, explosives, and vehicle damage.	*During VR:* HRV, distress *About VR*: cortisol, distress, dissociation	No difference in HRV or cortisol levels was found between PTSD and non-PTSD veterans. Higher levels of distress and dissociation in VR were found in PTSD compared to non-PTSD.
**Eating disorders**					
Provenzano et al. (23), Italy	20 anorexia nervosa (100%♀) 20 HC (100%♀)	12	***Body image:*** Lay on a deck chair while full-body illusion is induced 3 times: first in a self-selected best-resembling avatar, then in a 15% thinner and 15% heavier avatar. Before and after each embodiment, subjects choose the avatar best resembling their real and ideal body out of 28 avatars.	*During VR:* body dissatisfaction (choose real and ideal VR body), VAS emotional response (from very negative to very positive), VAS similarity rating, VAS attractiveness rating	AN associated 15% thinner avatars with increased attractiveness, in contrast, HC associated this with a decrease in attractiveness. AN felt more negative emotions after the 15% heavier avatar than the 15% thinner. HC felt more negative emotions after the thinner avatar than the heavier. In AN, emotional response correlated large with two established measures on body shape concerns (BSQ), and body uneasiness (BUT-GSI).
Porras-Garcia et al. (24), Spain	30 anorexia nervosa (100%♀) 43 HC (100%♀)	6	***Body image and biomarker:*** After full-body illusion in a self-resembling avatar in terms of body size, observe the body from a first-person perspective and look at your virtual self in the mirror.	*During VR:* eye-tracking, VAS body anxiety, VAS fear gaining weight	AN had higher fear of gaining weight, body anxiety than HC. AN directed gaze more to the stomach, hips, and thighs than HC. VAS body anxiety and VAS fear of gaining weight correlated medium-large to all eating disorder measures (EDI, PASTAS, BIAS, BAS).
Summers et al. (25), USA	25 body dysmorphic disorder (72%♀) 25 HC (72%♀)	30	***Appearance related bias** (360°):* Watch 13 scenes with ambiguous social cues, e.g., people looking toward the subject in a doctor's office.	*About VR:* likelihood of a benign and appearance-related threat scene interpretation, distress, VAS peak perceived threat, VAS urge to avoid, VAS urge to check appearance	BDD had greater threat interpretation biases, distress, perceived threat, urge to check, urge to avoid in response to VR than HC. These group differences in interpretation bias were replicated with two established measures (BDD-SWAP, IQ).
**Psychotic disorders**					
Shaikh et al. (26), UK	64 ultra-high risk for psychosis (41%♀) 42 HC (53%♀)	4	***Paranoia:*** Enter a tube train and disembark at the second stop.	*About VR*: state paranoia (SSPS)	State paranoia was higher in UHR than HC. The PQ paranoia subscale predicted state paranoia in VR. The perceived ethnic discrimination questionnaire did not predict state paranoia in VR.
Veling et al. (27), The Netherlands	55 psychotic disorder (24%♀) 20 ultra-high risk for psychosis (65%♀) 42 siblings (45%♀) 53 HC (53%♀)	20	***Social stress and paranoia:*** During 5 bar visits, find the five avatars with numbered shirts and remember the highest number and gender of that avatar. The number of social stressors differed each bar visit, by manipulating crowding, and avatars' hostility and ethnicity.	*About VR*: VAS distress, state paranoia (SSPS)	Paranoia and distress increased with the number of social stressors in the bar. UHR and patients with psychotic disorders experienced more distress in VR than HC and siblings. UHR had higher state paranoia in VR than HC. Paranoia and distress in VR was predicted by paranoid thoughts (GPTS), social anxiety (SIAS), positive, negative and depressive symptoms (CAPE).
Geraets et al. (18), The Netherlands	50 psychotic disorder (24%♀) 19 ultra-high risk for psychosis (65%♀) 40 siblings (45%♀) 47 HC (53%♀) S*ubsample Veling et al. (27)*	20	***Social stress and interpersonal distance:** Same as Veling et al. (27) above*.	*During VR*: interpersonal distance *About VR*: VAS distress, state paranoia (SSPS)	No difference in interpersonal distance between groups was found. Interpersonal distance was positively associated with social anxiety (SIAS) and distress in VR, but not with state paranoia, depressive, positive or negative symptoms.
Hesse et al. (28), Germany	31 schizophrenia/ schizoaffective disorder (29%♀) 20 HC (35%♀)	22	***Social stress**:* Approach five colleagues in an office to ask for help (task 1) and to collect money for a present (task 2). Pre-recorded reactions are given that are neutral or negative (deny help/don't give money).	*About VR:* state paranoia (SSPS)	Patients had higher state paranoia than HC. State paranoia in VR correlated medium-large with delusions measured with established measures (PSYRATS, PANNS) in patients. Trends indicated heightened paranoia in patients after social rejection.
Dietrichkeit et al. (29), Germany	39 schizophrenia (38%♀) 20 HC (50%♀)	20	***Memory bias and cognitive insight:*** During 4 tasks, explore a street and metro station with 12 pedestrians showing happy, neutral, or angry expressions, and explore a beach and campground.	*About VR:* avatar and object memory (emotion, items, location), memory confidence	Patients did not show poorer recognition performance than HC in most categories (object, emotion, and location recognition). Only in recognizing faces, patients made more failures than HC. Patients did not have more false memories than HC.
**Anxiety disorders**					
Diemer et al. (30), Germany	40 acrophobia (75%♀) 40 HC (70%♀)	10	***Biomarker anxiety:*** Look down from a 14m high roof and approach the edge while standing on a wooden board. Then, look down into the street 5x and look straight ahead 5x.	*During VR:* HR, skin conductance, VAS anxiety *During VR:* cortisol	Anxiety was higher in AD than HC. AD and HC both showed heightened HR and skin conductance. Only when looking down, AD had a higher HR increase than HC. No difference in cortisol reaction was found between groups. In AD, the Acrophobia Questionnaire correlated medium with VAS anxiety but not with HR or skin conductance.
Breuninger et al. (31), Germany	21 agoraphobia/panic disorder (71%♀) 27 HC (63%♀)	20	***Biomarker anxiety**:* Move out of a parking garage to meet friends. Then, take the elevator to return, something unexpected might happen. Subjects hear an explosion (stressor), screaming, smell smoke, see the car and truck on fire, and a person trapped. Subjects can press alarms, use fire extinguishers or move. Harm is indicated by coughing and red flashes when moving in smoke or flames. It ended when leaving the garage. Before and after, subjects engaged in VR relaxation.	*During VR*: HR, HRV, skin conductance *About VR:* VAS subjective HR, VAS subjective sweat perception, VAS anxiety, state emotion regulation strategies (ERQ-S)	No difference in anxiety, skin conductance, or emotion regulation strategies was found between groups. Following the stressor, less HR increase was seen in AD than HC. HRV declined more in HC than AD after the stressor. Subjective perception of HR and sweat was stronger in AD than HC.
Kishimoto and Ding (32), China	26 social anxiety disorder (50%♀) 26 HC (46%♀)	6	***Biomarker anxiety and social stress:*** Give 2 speeches in a room with 6 people. The audience provides mild ambiguous or intense negative feedback.	*During VR:* HR *About VR*: VAS discomfort	No difference in HR was found between groups. SAD had higher discomfort levels than HC In the mild ambiguous feedback condition, but not in the negative feedback condition.
Felnhofer et al. (33), Austria	12 social anxiety disorder (75%♀) 18 HC (66%♀)	15	***Biomarker anxiety:*** Task 1: order a drink from a waiter in the café. Task 2: respond to a stranger's request. Task 3: react to being offered a false drink. Responses from preprogrammed reactions are given.	*During VR:* HR *About VR:* anxiety (STAI-S)	SAD reported higher anxiety levels but in general similar responses in HR to the tasks than HC.
Kim et al. (34), Korea	79 social anxiety disorder (49%♀) 51 HC (47%♀)	12	***Gaze behavior:*** Give 6 speeches in a room with 7 people. Some take notes and look at the speaker, others show signs of distraction. 2 speeches have to be prepared, and 4 performed directly after receiving the topic.	*During VR:* eye-tracking *About VR:* speech performance	HC had better researcher-rated speech performance than SAD. SAD directed less gaze to the audience than HC. HC looked more at the audience when presenting self-related than general topics, no difference was found in SAD. In SAD, while presenting general topics, audience gaze correlated small-medium with only two anxiety measures (STAI-State, STAI-Trait), during self-related topics gaze related small-medium with fear of negative evaluation. No significant correlations were found in other anxiety measures (SIAS, LSAS, SPS) or in HC.
Guitard et al. (35), Canada	28 generalized anxiety disorder (86%♀)	5	***Anxiety:*** Task 1: walk through an empty virtual room (neutral). Task 2 (stressor): visit one place based on the main worry theme: a) an emergency room, b) an apartment overhearing a conversation on an accident, seeing a rock thrown at the window and someone outside roughed up, and hear an answering machine message on the personal worry theme, or c) a student room with bills and talks about failing.	*About VR*: anxiety (STAI-S), negative affect (PANAS)	Anxiety was higher after both the VR stressor task and after imagination of a personalized catastrophic scenario, as compared to the neutral VR task. Negative affect did not increase after the VR task or imagination.
Holmberg et al. (36), Denmark	10 social anxiety disorder (10%%♀) 9 HC (11%♀)	12	***Anxiety*** (360°): View three shopping scenes graduating in stressors: 1) moving through the shopping center, 2) walk around a shop, being welcomed, standing in a short queue and receive a gift card, 3) follows the same script, but with a longer queue, more bystanders, and negative statements about the personnel/service.	*During VR:* VAS anxiety	Anxiety of SAD was higher in two videos compared to HC.
**Addiction disorders**					
Wang et al. (37), China	61 methamphetamine use disorder (0%♀) 45 HC (0%♀)	8	***Biomarker craving** (360°):* View 6 people using methamphetamine, who also invite the subject to take methamphetamine.	*During VR:* HRV *About VR:* VAS craving, VAS likelihood of using if available	The VR task induced higher HRV in meth-dependent participants than HC. In meth-dependent participants, HRV changes were positively correlated with VR-induced cravings.
Ding et al. (38), China	333 methamphetamine-dependent (0%♀) 332 HC (0%♀)	15	***Biomarker craving:*** View a neutral grassland and relax. View 3 scenarios in a bedroom, car, and karaoke bar where avatars use methamphetamine and drug paraphernalia. Participants can pick up drug paraphernalia and virtually use them. Auditory cues appear when drugs are taken.	*During VR*: EEG, skin conductance	Meth-dependent participants had lower skin conductance, EEG power in delta, and alpha bands than HC. EEG power of beta band and gamma-band was higher in meth-dependent participants than HC. A logistic regression algorithm showed high specificity and sensitivity in distinguishing meth-dependent participants from HC using skin conductance and EEG data.
Ghita et al. (39), Spain	13 alcohol use disorder (38%♀) 14 social drinkers (86	13	***Craving:*** In ALCO-VR subjects are exposed to 5 favorite alcoholic drinks and water in 5 environments (neutral white room, bar, pub, restaurant, and home), from the least to most craving inducing drinks and environments. Subjects approach, hold, and observe each drink 20s with controllers. Olfactory stimuli are applied.	*During*: VAS craving, VAS anxiety *About VR:* craving (MACS-VR), anxiety (STAI-S)	ALCO-VR elicited stronger anxiety and alcohol craving in AUD than social drinkers. Anxiety response differentiated AUD and social drinkers better than cue-induced craving. In AUD, large correlations between craving and anxiety in VR with an established alcohol disorder measure (AUDIT) were found. Also, craving in VR was strongly correlated with trait anxiety (STAI).
Bouchard et al. (40), Canada	34 gambling disorder (35%♀)	14	***Craving and identifying risk situations:*** Visit and gamble in a casino and a bar with video lottery machines. Socratic questioning was applied during VR to evoke at-risk situations, emotions, and dysfunctional thoughts.	*During VR:* verbalize thoughts *About VR*: VAS craving	VR immersion helped identify more high-risk situations and twice as many dysfunctional thoughts (non-significant) than imagining a gambling session.
Chrétien et al. (41), Canada	29 gambling disorder (48%♀)	14	***Craving and identifying risk situations**: Same as Bouchard et al. above (40)*.	*During VR:* verbalize thoughts	More gambling-specific thoughts and more categories of thoughts were verbalized in the VR condition than during imagination. No difference between VR and imagination was found regarding the number of verbalized addiction-related thoughts.
Shin et al. (42), Korea	34 internet gaming disorder (0%♀) 30 HC (0%♀)	25	***Craving and refusal:*** Task 1) enter an internet café. Task 2) Observe peers talking about gaming and World Championships. Task 3) reject or accept two game invitations by peers. Task 4) refuse invitations to play a game.	*During VR:* VAS craving, game acceptance rate	IGD had higher craving than HC. Entering the café and being invited to game resulted in higher craving than observing a conversation in both groups. IGD showed a higher game acceptance rate than HC. Correlation analysis with established measures (modified IAT, IAT) showed mixed results.
Lee et al. (43), Korea	23 internet gaming disorder (0%♀) 29 HC (0%♀)	10	***Identify leisure time:*** Select rooms, explore and select leisure activities by choosing objects and determine how likely it is that they do the activity in daily life. Day and night-time activities were assessed separately.	*During VR:* leisure activities	IGD had fewer leisure activities and preferred game/digital activities compared to HC.

*All VR was simulated unless 360° is mentioned. All mentioned differences concern statistical significant differences only, unless mentioned otherwise. The duration of the tasks in minutes is an approximation.*

*AD, Anxiety disorder; AUD, alcohol use disorder; AUDIT, Alcohol Use Disorder Identification Test; AN, anorexia nervosa; BAS, Body Appreciation Scale; BDD, body dysmorphic disorder; BDD-WSAP, Word–Sentence Association Paradigm modified for BDD; BIAS, Body Image Assessment Test; BSQ, Body Shape Questionnaire; BUT-GSI, Body Uneasiness Test; CAPE, Community Assessment of Psychic Experiences; EDI-3, Eating Disorder Inventory; ERQ-S, Emotion Regulation Questionnaire-State; GPTS, Green Paranoid Thought Scale; HC, healthy controls; HR, heart rate; HRV, heart rate variability; IGD, internet gaming disorder; IQ, Interpretations Questionnaire; LSAS, Liebowitz Social Anxiety Scale; MACS-VR, Multidimensional Alcohol Craving Scale adjusted for VR; PANAS, Negative Affect Scale of the Positive and Negative Affect Schedule; PASTAS, Physical Appearance State and Trait Anxiety Scale; PQ, Prodromal Questionnaire; SAD, social anxiety disorder; SIAS, Social Interaction Anxiety Scale; SPS, Social Phobia Scale; SSPS, Social State Paranoia Scale; STAI-S, State-Trait-Anxiety-Inventory-State; STAI, Trait Anxiety Inventory; UHR, ultra-high risk for psychosis; VAS, verbal/visual analog scale*.

**Table 2:** Immersive VR-assisted psychiatric assessment studies EPHPP quality rating.

	**Selection bias**	**Study design**	**Confounder**	**Blinding**	**Data collection method**	**Withdrawals and dropouts**	
	**A**	**B**	**C**	**D**	**E**	**F**	**Global rating**
Camacho-Conde et al. (19)	3	2	3	2	1	2	2
Fromberger et al. (20)	2	2	2	2	3	3	3
van't Wout et al. (21)	3	2	3	2	2	3	3
Ridout et al. (17)	3	2	3	2	2	2	3
Malta et al. (22)	2	2	3	2	2	3	3
Provenzano et al. (23)	2	2	3	2	1	3	2
Porras-Garcia et al. (24)	2	2	3	1	3	2	2
Summers et al. (25)	2	2	3	2	2	3	3
Shaikh et al. (26)	2	2	3	2	3	2	3
Veling et al. (27)	2	2	2	2	2	3	3
Geraets et al. (18)	2	2	2	2	2	3	3
Hesse et al. (28)	2	2	3	3	2	3	3
Dietrichkeit et al. (29)	2	2	2	2	3	3	3
Diemer et al. (30)	1	2	3	2	3	3	2
Breuninger et al. (31)	2	2	3	2	3	3	3
Kim et al. (34)	2	2	3	3	2	3	3
Kishimoto and Ding (32)	2	2	3	2	2	3	3
Felnhofer et al. (33)	1	2	3	2	2	2	2
Guitard et al. (35)	2	2	3	2	3	3	3
Holmberg et al. (36)	2	2	2	2	1	3	2
Wang et al. (37)	2	2	3	1	2	3	2
Ding et al. (38)	2	2	3	2	2	3	3
Ghita et al. (39)	2	2	3	2	2	3	3
Bouchard et al. (40)	3	3	2	2	1	2	2
Chrétien et al. (41)	3	3	3	2	1	3	2
Shin et al. (42)	2	2	3	2	2	3	3
Lee et al. (43)	2	2	2	2	1	3	2

## Discussion

With this systematic review, we aimed to provide an overview of recent (2016–2020) publications on immersive VR-assisted psychiatric assessment. In total, 27 studies were identified, however, the goals of assessment differed significantly, ranging from assessment of symptoms for diagnostics, to give direction to therapy, search for biomarkers, or to be used in research for gaining increased understanding of a disorder. First, the findings per diagnostic group will be discussed.

### Recent VR-Assisted Psychiatric Assessment Studies

#### Developmental Disorders

Research on VR assessment in adults with developmental disorders was very limited. A single study on ADHD was identified, comparing young, mostly adult, males with and without ADHD (19). Similar to research in children, this study concerned a VR-assisted neuropsychological test (Nesplora Aquarium test) that continues the wave of 2D computerized continuous performance tasks for assessment of attention, working memory, and processing speed (12). No studies were identified on autism spectrum disorders (ASD). The objective of using new technologies for assessment is often to make a diagnosis as early as possible. As ASD is a developmental disorder this may explain the lack of research in adults. However, recent reviews including children with ASD show a similar gap in knowledge on the utility of VR for assessment (44, 45).

#### Pedophilic Disorder

One feasibility study has been conducted in the field of forensic psychiatry since the first research by Renaud et al. in offenders with a pedophilic disorder (46, 47). In this study, Fromberger and colleagues tested, in pedophilic sex offenders, whether coping behaviors in VR risk scenarios—encountering children asking for help in a supermarket—can provide additional information for risk assessments (20). Many offenders behaved differently in VR than their own beliefs about desired responses and coping strategies they had focused on in treatment. Also, therapists predicted offender's behavior wrongly 25% of the time. This indicates that VR-assisted assessments can add a new dimension to risk assessments by testing, in a safe environment, whether learned or desired strategies transfer to behavior. Especially in forensic settings, this can be useful as behavior assessments on risk situations in real life would be highly unethical. Furthermore, applying VR-assisted risk assessments could in part counteract the social desirability which is common among offenders self-reporting on their own intended behaviors related to offending. However, the authors indicated that further development of the task was needed, in terms of response possibilities and the creation of scenarios with fewer moral dilemmas.

#### PTSD

VR-assisted assessment in PTSD has mainly focused on differentiating between veterans with and without PTSD by using physiological measures during VR exposure to combat situations (17, 21, 22). However, only differences in skin conductance level (21), but not in heart rate variability (HRV) (17, 22) nor cortisol levels (22), were found between groups. The difference in skin conductance was specific for combat situations, in a classroom environment no such difference was found. Interestingly, the skin conductance (21) and HRV (17) research concerned the same sample, suggesting that skin conductance measures may be more sensitive. Similarly, van Bennekom et al. also identified one study in PTSD, also showing heightened skin conductance in PTSD veterans compared to healthy controls (48).

#### Eating Disorders

Whereas, previous assessment research in eating disorders has mainly focused on the reaction of patients to virtual food (12), three recent studies have focused on body image and appearance-related biases. Two studies on anorexia nervosa used the technique of body illusion or embodiment (23, 24). Embodiment refers to the illusion that a virtual life-sized body, seen from a first-person perspective, is experienced as one's own body (49, 50). Medium to large correlations were found between established self-report questionnaires on eating disorders and single item VR VAS measures on fear of gaining weight, body anxiety, and emotional responses (23, 24). Further, anorexia patients felt more negative emotions after being embodied in a heavier body than a thinner one than their own. Healthy controls showed opposite reactions; feeling more negative in thinner bodies than heavier ones (23). Furthermore, clear distinctions between controls and patients were found with eye-tracking; anorexia patients directed their gaze to different parts of the body than healthy people (24). Summers et al. used VR differently, showing ambivalent 360° videos (e.g., overhearing a conversation about someone else's appearance) to patients with a body dysmorphic disorder and focused on interpretation bias (25). Also here, differences to controls were found in interpretation and emotional reactions to the VR scenes, consistent with well-established measures. The authors concluded that VR assessment may help to provide more nuanced treatment targets.

#### Psychotic Disorders

Following previous work on psychosis (10, 12), recent VR-assisted assessments have been done to measure paranoia, social stress, and memory bias in patients with a psychotic disorder or at ultra-high risk for psychosis. Consistently paranoid thoughts, measured by the State Social Paranoia Scale (51) were triggered in patients (18, 26–28, 51). Important to note, the VR paranoia tasks of these studies differed in content; Shaikh and colleagues exposed people to a neutral tube train ride (26), whereas the other studies exposed people to situations differing in the amount of social stress (e.g., being in a bar with neutral or hostile looking people (18, 27), or asking help from colleagues and receiving neutral or negative reactions (28).

#### Anxiety Disorders

In the domain of anxiety, VR has mainly been used to investigate feelings of anxiety and physiological responses during general stressful and social interaction situations (e.g., where participants had to give a speech or respond to a stranger in a bar). All studies reported that patients with an anxiety disorder consistently showed higher levels of subjectively experienced anxiety, except for Breuninger et al. who found that similar levels of anxiety were provoked during a VR emergency situation (fire in a parking garage). Concerning physiological measures, skin conductance (30, 31), cortisol (31), heart rate (30–33), and eye-tracking (34) were measured. No differences in skin conductance, nor cortisol reactions, were found between patients and controls. Regarding heart rate measures, reactions in VR environments tended to be similar in controls and patients (30, 32, 33), with two studies showing some differences when zooming in on specific stressor moments (30, 33). Interestingly, Breuninger et al. showed that the subjective perception of sweat and heart rate differed strongly between patients and controls, in contrast to the actual physiological measures. These findings contrast to the Bennekom et al. review (12), which concluded that most physiological measures were in accordance with subjective anxiety measures.

#### Addictive Disorders

Seven articles were found on addictive disorders, ranging from methamphetamine and alcohol disorders to gambling and gaming disorders, mainly studying craving. Many different types of designs were used, with 5/7 studies having a control group, and some measuring only biological markers. Differences between patients and controls were found in both craving and anxiety in alcohol disorder (39) and internet gaming disorder (42), as well as differences in physiological reactions for methamphetamine disorder (HRV and EEG) (37, 38). Two studies used VR in the context of treatment (40, 41). Engaging in virtual gambling was used in both studies in combination with Socratic questioning to identify risk situations, where participants had to verbalize their thoughts during the VR exposure. The researchers found that patients reported more dysfunctional thoughts and more categories of thoughts during VR exposure than during mental imagery. Based on this, the visual component of VR may be helpful to identify risk situations and treatment targets for patients with addictive disorders.

### VR Task Characteristics

Task characteristics differed substantially on several factors, such as the type of VR used, level of social and object interaction, level of personalization, and modalities used e.g., smell and touch. No ideal task characteristics seem to be identifiable, as e.g., the amount of interaction and personalization needed seems to vary strongly with the specific goal and purpose of the assessment. Three studies used 360° videos to assess anxiety, craving, and interpretation bias in body dysmorphic disorder. Although 360° videos limit interaction and personalization, they do offer standardized, easy, and cheap applications. In four of the 27 studies, some form of personalization was applied to the VR program. In two studies, self-resembling avatars were made for body image assessment (23, 24). Further, in the research of Fromberger et al., virtual child characters were selected before the assessment based on the sexual offender's level of attraction to them (20). Guitard et al. (35) personalized the task based on the main worry theme of a person. Three anxiety-inducing situations were available to choose from which fitted the worry theme best (an emergency room, apartment, or a student room scenario), and in one of them, people would hear a message on an answering machine based on their personal worry theme.

### The Future of VR-Assisted Psychiatric Assessments

In line with the earlier reviews, all VR tasks were able to detect some difference between patient groups with a psychiatric disorder and healthy control groups, with regard to self-report measures (10, 12). However, the road from research to clinical practice, to apply VR for diagnostic purposes and provide direction for psychiatric treatment, seems still substantial. Most of the tasks applied so far are lacking reliability and validity, and no test-retest designs were identified. In general, samples were small, with 60% including patient groups of maximally 30 people. Further, concerning behavioral tasks, a comparison between VR and real-world behavior is lacking. In order to answer the question as to how VR can affect the ecological validity of psychiatric assessments, such studies are needed.

Concerning physiological measures, findings were less convincing than studies including self-report measures. In several studies, similar responses were observed in patients and controls. E.g., veterans with PTSD did not differ in HRV or cortisol from veterans without PTSD during VR combat scenarios, findings on heart rate in anxiety-inducing situations were mixed, and interpersonal distance regulation in response to social stressors did not differ between people with different psychosis liability. These findings pose questions about the usefulness of physiological measures for psychiatric assessment, despite their potential as an “objective” measure. When physiological measures are used during VR, this is done to measure real-time reactions in response to scenarios or specific stimuli. During VR, people often move, see multiple things which contrast strongly to controlled laboratory settings in which physiological measures are usually performed, which may induce error in the instrument readings. Possibly baseline measures—and not reactive measures to complex stimuli such as VR– may be more valuable in psychiatric assessments.

An important conclusion that may be drawn from the current study is that a knowledge gap still exists on the value and possible benefits of VR-assisted assessments as an addition to current psychiatric diagnostic processes. In coming research, it would be relevant to study what additional information VR-assisted assessments can provide next to other assessments, instead of evaluating VR as a substitute to more conventional psychiatric assessments. Fromberger et al. made a first step in this with research on pedophilic offenders (20). Furthermore, in clinical practice, assessments can also be intertwined with treatment. For example, during a VR-based CBT intervention, safety behaviors were continuously observed and paranoid thoughts were explored and emphasized on (52). Similarly, Riches et al. assessed paranoid ideation with interviews after VR exposure in a non-clinical high trait paranoia group, and concluded that themes identified through this assessment could help inform target areas for treatment (53). This form of assessment was also explored by the research group of Bouchard (40, 41), who analyzed verbalized thoughts of patients prompted by Socratic questioning during VR exposure to gambling situations. Thus, the potential of VR in combined assessment and treatment should be further investigated.

One other objective pursued by applying new technologies such as VR for assessment is to find alternative ways of classifying patients. This has been specifically addressed in frameworks investigating mental disorders, such as the Research Domain Criteria (RDoC), where it is argued that the understanding of mental disorders through observable behaviors and self-reports should be complemented by other, so-called, units of analysis that are objectively collected e.g., with (neuro)physiological measures (54). This may allow obtaining new patient stratifications that better explain their response to treatment. Thus, for future research it is not only of relevance to investigate the relationship between a VR and an established measures but also to provide new measures (biomarkers) that could better explain the different phenotypes related to mental health.

Future research may also take advantage of qualitative approaches, e.g., with a more hypothesis-generating character. VR tasks can trigger feelings and cognitions which can be discussed during or after the VR task. Such an approach may be less objective but could turn out to be highly relevant in clinical practice. Also, other (neuro)physiological measures such as pupil dilation and saccadic eye movements, previously suggested as biomarkers for mental disorders, should be investigated using VR eye-tracking technology, in line with suggestions by the RDoC framework (55, 56). Future research should also further investigate the so-called Proteus effect (57). This effect shows that when using embodiment, characteristics of the embodied avatar can also influence an individual. E.g., people embodied in taller avatars have been found to behave more confidently during a negotiation task than people embodied in shorter avatars (57). Further, including larger groups will enable more profound investigations of the relationship between established psychiatric assessment measures and VR-assisted measures. Finally, coming research could benefit from the inclusion of participants on a spectrum from healthy controls to patients in clinical settings, and adopting a more transdiagnostic approach.

### Limitations

This review has several limitations. First, only studies that were published in English were included, possibly limiting the evidence-base to draw conclusions upon. Second, this review concerns a relatively new technique, resulting in rather small sample sizes in the reviewed studies. A third limitation concerns the definition of adult samples; studies were included if either an age cut-off of 18 years and older was used (this was the case in the majority of the studies) or if the mean age was 18 years or older. Herewith also some adolescents were included in some studies which is a limitation. Also, a great variety of virtual environments and tasks were used in the included studies. Furthermore, studies were mixed in the use of a patient and control group, and not all studies provided results on relations between the VR-measures and traditional assessment measures. Additionally, some studies concerned a combination of VR-assisted assessment and treatment (40, 41), which made abstraction of the measures more difficult. However, in clinical practice, assessment will often be used to guide a therapeutic intervention. Therefore, one could also state that such trials combining both assessment and treatment closely approach the actual use in clinical settings. Finally, we did not include conference proceedings, which presents a limitation in such a new field as the current, with rapid developments.

## Data Availability Statement

The original contributions presented in the study are included in the article/Supplementary Material, further inquiries can be directed to the corresponding author/s.

## Author Contributions

CG and MW designed the study. CG performed the literature search and wrote the first draft of the manuscript. CG and KS carried out the analysis of the results. All authors contributed to manuscript revision, read, and approved the submitted version.

## Funding

Region Kronoberg (grant number 961523) and the Swedish Research Council for Health, Working Life and Welfare (grant number 2018-01409) have provided funding for this research.

## Conflict of Interest

The authors declare that the research was conducted in the absence of any commercial or financial relationships that could be construed as a potential conflict of interest.

## Publisher's Note

All claims expressed in this article are solely those of the authors and do not necessarily represent those of their affiliated organizations, or those of the publisher, the editors and the reviewers. Any product that may be evaluated in this article, or claim that may be made by its manufacturer, is not guaranteed or endorsed by the publisher.
