# Supplementary material for: Use of Virtual Reality in Psychiatric Diagnostic Assessments: A Systematic Review
Source: Front Psychiatry. 2022 Feb 28;13:828410. doi: 10.3389/fpsyt.2022.828410 (PMC8918631; doi:10.3389/fpsyt.2022.828410)
Supplement: Supplementary file 1 [file Table_1.DOCX]

**Supplement 1**

**EMBASE** (Emtree search terms (/exp) are underlined)

(2016:py OR 2017:py OR 2018:py OR 2019:py OR 2020:py)

**AND** ('virtual reality'/exp OR 'virtual reality' OR 'VR technology')

**AND** ('diagnosis'/exp OR 'assessment of humans'/exp OR **'**clinical assessment tool**'**/exp OR **'**evaluation and follow-up**'**/exp OR assess* OR diagnos* OR evaluat* OR test* OR experiment*)

**AND** ('mental disease' OR 'mental disorder' OR psychiatr*

OR 'mood disorder'/exp OR depression OR bipolar OR 'mood disorder' OR mania

**OR** 'anxiety disorder'/exp OR anxiety OR phobia OR panic OR 'obsessive compulsive' OR 'post traumatic' OR PTSD
**OR** 'autism'/exp OR autism OR asperger

**OR** 'personality disorder'/exp OR 'personality disorder' OR psychopath*
**OR** 'forensic psychiatry' OR 'forensic psychology'

**OR** 'eating disorder'/exp OR 'anorexia' OR 'bulimia' OR 'binge eating'

**OR** psychosis/exp OR 'schizophrenia spectrum disorder'/exp OR schizophren* OR delusion OR hallucination
**OR** 'psychosexual disorder/exp' OR 'sexual behavior'/exp OR 'sexual'

**OR** 'behavior disorder'/exp OR 'impulse control' OR 'disruptive disorder' OR 'conduct disorder' OR 'attention deficit' OR ADHD

**OR** addiction/exp OR 'drug dependence' OR ’substance dependence' OR 'substance disorder' OR abuse OR alcohol OR cannabis OR tobacco OR amphetamine OR hallucinogens OR opioid OR heroin OR stimulant OR sedative)

**PsycInfo** (Thesaurus terms are underlined)

**Limiters**: 2016-2020, Apply equivalent subjects ON -> **YES**, related words -> NO

(virtual reality OR VR technology)
**AND** (diagnosis OR diagnos* OR assess* OR evaluat* OR test* OR experiment*)
**AND** (“mental disorders” OR psychiatr*
**OR** “affective disorders” OR “mood disorders” OR bipolar OR depression OR mania
**OR** “anxiety disorders” OR anxiety OR “posttraumatic stress” OR “post traumatic” OR PTSD OR phobia OR panic OR “obsessive compulsive”

**OR** “autism spectrum disorders” OR autism OR Asperger

**OR** “personality disorders” OR psychopath*
**OR** forensic psychology OR forensic psychiatry

**OR** eating disorders OR anorexia OR bulimia OR binge eating

**OR** psychosis OR psychotic OR schizophren* OR delusion* OR hallucination*
**OR** psychosexual behavior OR sexual OR paraphil*

**OR** “behavior disorders” OR “attention deficit” OR ADHD OR “conduct disorder” OR “impulse control” OR disruptive disorder

**OR** substance Related and Addictive Disorders OR substance disorders OR drug dependence OR substance dependence OR abuse OR alcohol OR cannabis OR tobacco OR amphetamine* OR opioid* OR heroin OR hallucinogen* OR stimulant* OR sedative*)

**PUBMED** (MeSH terms are underlined)

("2016/01/01"[Date - Publication] : "2020/12/31"[Date - Publication] )

**AND** (Virtual reality OR “VR technology”)

**AND** (diagnosis OR outcome assessment OR “behavioral symptoms” OR assess* OR diagnos* OR evaluat* OR test* OR experiment*)

**AND** (“mental disorders” OR psychiatr*
**OR** depression OR “mood disorder” OR mania OR bipolar

**OR** anxiety OR panic OR phobia OR “obsessive compulsive” OR “post traumatic” OR PTSD

**OR** autism OR Asperger

**OR** “personality disorder” OR psychopath*

**OR** forensic psychiatry OR forensic psychology

**OR** feeding and eating disorders OR anorexia OR bulimia OR binge eating

**OR** psychotic disorders OR psychosis OR schizophren* OR delusion* OR hallucination*

**OR** sexual dysfunction OR paraphilic disorders OR sexual

**OR** disruptive, impulse control, and conduct disorders OR attention deficit and disruptive behavior disorders OR “conduct disorder” OR “impulse control” OR disruptive disorder OR ADHD OR “attention deficit”

**OR** substance-related disorders OR substance disorder OR drug dependence OR substance dependence

OR abuse OR alcohol OR cannabis OR tobacco OR amphetamine OR opioid OR heroin OR hallucinogens OR stimulants OR sedative)

**Web of Science**

**Timespan**: 2016-2020

TS=((virtual reality OR “VR technology”)
**AND** (behavio$ral symptom OR assess* OR diagnos* OR evaluation* OR test* OR experiment*)

**AND** (“mental disorder” OR “mental disorders” OR “mental disease” OR psychiatr*
**OR** “affective disorder” OR “mood disorder” OR bipolar OR depression OR mania
**OR** anxiety OR posttraumatic OR “post traumatic” OR PTSD OR phobia OR obsessive compulsive OR panic

**OR** autism OR Asperger

**OR** “personality disorder” OR psychopath*
**OR** forensic psychology OR forensic psychiatry

**OR** eating disorder OR anorexia OR bulimia OR binge eating

**OR** psychotic OR psychosis OR schizophren* OR delusion* OR hallucination*
**OR** psychosexual OR paraphil* OR sexual **OR** “behavior disorder” OR “attention deficit” OR ADHD OR “conduct disorder” OR “impulse control” OR disruptive disorder

**OR** addiction OR addictive disorder OR substance disorder OR drug dependence OR substance dependence OR abuse OR alcohol OR cannabis OR tobacco OR amphetamine* OR opioid* OR heroin OR hallucinogen* OR stimulant* OR sedative*))
